# Sodium butyrate and tributyrin induce *in vivo* growth inhibition and apoptosis in human prostate cancer

**DOI:** 10.1038/sj.bjc.6601510

**Published:** 2004-01-20

**Authors:** R Kuefer, M D Hofer, V Altug, C Zorn, F Genze, K Kunzi-Rapp, R E Hautmann, J E Gschwend

**Affiliations:** 1 Department of Urology, University of Ulm, Prittwitz-Strasse 43, 89075 Ulm, Germany; 2 Institute for Lasertechnology in Medicine, University of Ulm, 89081 Ulm, Germany; 3 Department of Pathology, Brigham & Women's Hospital, Boston, MA 02115, USA

**Keywords:** sodium butyrate, tributyrin, HDAC, prostate cancer, *in vivo*, apoptosis

## Abstract

Histone deacetylase inhibitors (HDACs) are known to exhibit antiproliferative effects on various carcinoma cells. In this study, the *in vivo* efficiency of two HDACs, sodium butyrate and tributyrin, on prostate cancer growth inhibition were investigated. To gain an insight into the possible underlying pathways, cell culture experiments were performed focusing on the expression of p21, Rb and c-myc. For *in vivo* testing, prostate cancer cell lines (PC3 and TSU-Pr1) were seeded on the chorioallantois membrane (CAM) and implanted in a xenograft model using nude mice. Standard Western blot analysis was performed for protein expression of p21, Rb and c-myc in HDAC-treated *vs* untreated prostate cancer cells. Both sodium butyrate and tributyrin had a considerable treatment effect on microtumours on the chicken egg at already very low concentrations of 0.1 mM. Tributyrin-treated tumours showed the strongest effect with 38% apoptotic nuclei in the prostate cancer cell line PC3. In the mouse model, there was almost no difference between sodium butyrate and tributyrin. In untreated animals the tumours were almost double the size 4 weeks after implantation. Tumours of the treatment groups had a significantly lower percentage of Ki-67-positive-stained nuclei. As demonstrated by Western blot analysis, these effects seem to be independent of p53 status and a pathway via p21–Rb–c-myc is possibly involved. In this study we have demonstrated a substantial *in vivo* treatment effect, which can be induced by the application of sodium butyrate or the orally applicable tributyrin in human prostate cancer. The given results may provide the rationale to apply these drugs in well-controlled clinical trials in patients being at high risk of recurrence after specific therapy or in patients with locally or distant advanced prostate cancer.

Prostate cancer has the highest incidence and is the second leading cause of cancer deaths among men in the United States (Jemal *et al*, 2002). With a response rate of about 80%, androgen ablation induces apoptosis of normal prostate epithelial cells as well as regression of early-stage prostate cancer. However, progression to androgen independence occurs over time, so that this treatment option is not curative (Samson *et al*, 2002). Conventional chemotherapy has not sufficiently been proven to be effective either, especially in androgen-independent prostate cancer, and is limited by toxicity that is often aggravated by comorbidity of the elderly prostate cancer patients (Yagoda and Petrylak, 1993). Promising results of a single agent have so far been reported only by the usage of Docetaxel (Taxotere) (Picus and Schultz, 1999). There is a tremendous need to identify substances suitable for the prevention of progression and as a means for a better cancer control in advanced prostate cancer cases.

Agents that are active against prostate cancer alter cell growth and differentiation by modifying the expression of genes or gene products that regulate cell shape, function, adhesion and communication. Such compounds are for example butyrates, members of the group of histone deacetylase inhibitors (HDACs) (Gleave *et al*, 1998). Butyrates are naturally occurring short-chain fatty acids leading to differentiation of numerous cell types (Chen and Breitman, 1994). The potential clinical utility of butyric acid is mainly limited by a short half-life *in vivo* as plasma concentrations decrease within minutes below the concentration needed for effects *in vitro* (Miller *et al*, 1987). To circumvent the problem of fast metabolism of butyrate monomers, analogues have been tested (Planchon *et al*, 1993). Tributyrin is a readily available trimer of butyric acid and is cleaved intracellular by lipases into three molecules of butyric acid. It has been shown that tributyrin can induce differentiation in human myeloid leukaemia cells, murine erythroleukaemia cells (Chen and Breitman, 1994), MCF-7 mammary carcinoma cells (Heerdt *et al*, 1999) and HT-29 colon cancer cells (Schroder *et al*, 1998). We have previously demonstrated that butyrates strongly induce *in vitro* growth inhibition and apoptosis in different human prostate cancer cell lines (Maier *et al*, 2000).

The objective of the present study was to determine whether *in vivo* prostate cancer tumour growth and progression are delayed under treatment with the butyrates sodium-butyrate and tributyrin. Testing was performed *in vivo* on the chorioallantois membrane (CAM) of fertilised chicken eggs (Kunzi-Rapp *et al*, 2001). For confirmation, a nude mouse model was explored with a study design of multiple dosing over 4 weeks. Immunohistochemistry for documentation of treatment-associated reduction of proliferation was performed. In addition, cell culture experiments were carried out to gain a further insight into the pathways possibly influenced by butyrates. Based on previous reports, the induction of p21, activation of the Rb protein and expression of c-myc were evaluated under treatment conditions with the HDACs (Blagosklonny *et al*, 1997; Claassen and Hann, 2000; Rashid *et al*, 2001).

## MATERIALS AND METHODS

### Cell culture and reagents

The LNCaP, PC3 and Tsu-Pr1 cell lines were obtained from American Type Culture Collection (Rockwell, MD, USA). All cell lines were cultured and maintained in 5% CO_2_ at 37°C in RPMI 1640 (Life Technologies, Eggenstein, Germany), supplemented with 10% heat-inactivated foetal calf serum, 2 mM L-glutamine, 100 U ml^−1^ penicillin G and 100 U ml^−1^ streptomycin (Life Technologies, Eggenstein, Germany). Sodium butyrate and tributyrin were obtained from Sigma-Aldrich Chemie GmbH (Deisenhofen, Germany). A stock solution of tributyrin was prepared using 100% ethanol. Sodium butyrate was dissolved in sterile water.

### Growth inhibition in cell culture

For determining cell proliferation, the viable cell numbers were counted using the Cell Proliferation Kit II (Roche Diagnostics GmbH, Mannheim, Germany) based on the XTT assay (Roehm *et al*, 1991). In brief, cells were grown in microtitre plates to 80% confluency. Cells were incubated with the given final concentration of the butyrates for 72 h. After incubation, 50 *μ*l of XTT labelling mixture was added to each well. The microtitre plate was incubated for another 4 h before measuring absorbance at 492 nm (reference wavelength 690 nm) of the samples using a microtitre plate reader (SLT spectra, Tecan GmbH, Crailsheim, Germany).

### Chorioallantois membrane

#### Preparation and procedure of grafting

Fertilised chicken eggs were incubated at 37.8°C and 60% relative humidity. On day 4 of incubation, implantation was prepared. Standard microbiology testing was used to rule out subclinical infections. The procedure of preparing the CAM was carried out as described previously (Kunzi-Rapp *et al*, 2001). Briefly, a part of the CAM was exposed by peeling a round aperture of 2 cm diameter. The resulting window was covered with a self-adhesive stripe and incubation was continued. At day 7 of incubation, a silicone ring with a thickness of 0.5 mm and an inner diameter of 6 mm was placed onto the membrane. The cells were seeded into the ring at a concentration of 5 × 10^5^ cells in 25 *μ*l serum-free RPMI 1640. Tumour growth and viability of the embryo were controlled daily by stereo microscopy.

#### *In vivo* apoptosis induction

Tumours were established as described 3 days after the inoculation of tumours on the CAM, sodium butyrate or tributyrin was administered intravenously (i.v.) into the CAM vessels using a 0.3 × 13 mm needle in a total volume of 100 *μ*l. The final *in vivo* concentration was calculated for a total blood volume of 2 cm^3^ and varied from 0.1 to 5.0 mM for tributyrin and sodium butyrate. At 48 h after application, the tumour, together with the CAM, was sampled for immunohistochemistry and apoptosis detection.

#### Quantitative assessment of apoptosis in implanted tumours

The detection of apoptotic cells was performed as described previously (Maier *et al*, 2000). Tumours were sampled 5 days after seeding and embedded in paraffin. Sections of 3 *μ*m were mounted on poly-L-lysine-coated slides. After deparaffinising and rehydration, slides were washed twice in PBS and treated with 0.1% H_2_O_2_ in PBS for 15 min. After repeated washing in dH_2_O and TdT buffer (30 mM Tris, pH 7.2, 140 mM sodium cacodylate, 1 mM cobalt chloride), slides were incubated with TdT–Biotin–dUTP mix (100 *μ*l TdT buffer, 30 U TdT, 0.5 *μ*l Biotin–dUTP; Boehringer Mannheim, Indianapolis, IN, USA) for 1 h at 37°C in a humid chamber. The reaction was stopped and unspecific binding was blocked by incubation in 2% BSA for 10 min at room temperature. Slides were incubated with a secondary antibody (ABC Vectastain, Vector Laboratories, Inc., Burlingame, CA, USA) for 30 min in a humid chamber at room temperature. Slides were washed and stained with 3-amino-9-ethylcarbazol (AEC). After washing in dH_2_O, slides were counterstained with haematoxylin, dehydrated and mounted. For determination of apoptosis, 500 cells were evaluated in representative fields at × 400 magnification, and apoptotic cells were calculated in percent of the total number of counted cells.

### Growth of cells in nude mice and procedure of treatment

Evaluation of drug-induced effects in the mouse model was performed using fast growing, hormone-independent PC3 and TSU-PR1 cells. Cells were maintained in culture as described and found to be free of mycoplasma contamination. Cells were harvested from tissue culture flasks after reaching semiconfluence. Before injection, cells were washed and resuspended in Dulbecco's phosphate-buffered saline (PBS). In all, 10^5^ cells were transplanted on both sides subcutaneously through 0.4 × 19 mm needles in projection of the scapulae of 7-week-old NMR/−nu/nu male mice with a mean weight of 30 g (Bomholtgard, Denmark). For each treatment protocol, six mice were used resulting in 12 tumours per setting. For intraperitoneal application, both sodium butyrate and tributyrin were dissolved in sterile saline. Treatment was started 24 h after implantation of the cells. During the first week, drug administration was performed on a daily basis for a calculated final plasma concentration of 10 mM. As the mice were estimated with a 2.4 ml blood volume, 24 mg of sodium butyrate and 7.9 mg of tributyrin were used (Egorin *et al*, 1999). From week 2 until the end of week 4, when the experiment was stopped, application was carried out every second day. As control, a mock-treated group with mice growing xenograft tumours and getting normal saline injections was observed. Tumour size was measured once a week and tumour volume was calculated according to the formula 1/2 × *L* × *W* × *H* in millimetres (Tomayko and Reynolds, 1989). The weight of all mice was measured weekly. All animal experiments were carried out with ethical committee approval (#631, Regierungspräsidium Tübingen, Germany) and met the standards as defined by the UKCCCR guidelines (Workman *et al*, 1998).

### Immunohistochemistry and quantitative assessment of KI-67 protein expression

Detection of the nuclear protein Ki-67 has been chosen, as it is preferentially expressed during the active phases of the cell cycle, but not in the G0-phase. Ki-67 is an accepted means for the detection of proliferating cells in paraffin-embedded tissue sections (Cattoretti *et al*, 1992; Gerdes *et al*, 1992; Mucci *et al*, 2000), and its expression has been shown to be associated with poor outcome in prostate cancer patients (Borre *et al*, 1998). Standard avidin–biotin complex immunohistochemistry was used for staining the animal tumours. Pretreatment was performed by exposure for 10 min in sodium citrate buffer in a microwave oven. The slides were then incubated sequentially with primary antibody (1 : 100 dilution, monoclonal mouse anti-human-Ki-67 antibody, DAKO Diagnostika, Hamburg, Germany), biotinylated secondary antibody, avidin–biotin complex and chromogenic substrate 3,3′-diaminobenzidine. Slides were evaluated for adequacy using a standard bright field microscope. Digital images were acquired and protein expression was scored as positive or negative using the MDS 5.8 system from Applied Imaging Corporation (Santa Clara, CA, USA). For objective evaluation, eight representative areas were defined. In the case of KI-67 protein expression, the system was optimised for nuclear staining, calculating the percentage of stained nuclei. Data were transferred to a spreadsheet for subsequent analysis.

### Western blotting

The expression of proteins was determined by Western blot analysis using specific primary antibodies for p21WAF1, Rb and c-myc (Oncogene, Cambridge, USA). Briefly, cells were lysed using RIPA solution (1% nonidet P-40, 0.5% sodium deoxycholate, 0.1% SDS, 100 *μ*g ml^−1^ phenylmethylsulphonylfluoride, 30 *μ*l ml^−1^ aprotinin, 50 *μ*g ml^−1^ leupeptin, in PBS, pH 7.4; chemicals were purchased from Sigma, Steinheim, Germany) at 4°C for 10 min. Lysates were centrifuged and the protein concentration of the supernatant was determined using the Bradford assay. Laemmli sample buffer at a ratio of 1 : 2 was mixed to the sample. Following electrophoresis, the proteins were transferred onto polyvinylidene difluoride membranes (Bio-Rad, Munich, Germany). Blocking was carried out with freshly prepared TBST plus 10% nonfat milk (20 mM Tris-HCl, pH 7.6, 137 mM NaCl, 0.1% Tween-20). After washing, the membrane was incubated for 1 h with primary antibodies diluted in TBST (100–1.500-fold). Appropriate horseradish peroxidase-conjugated secondary antibodies (Amersham, Buckinghamshire, UK) were incubated overnight. For detection, autoradiography using ECL was performed according to the manufacturer's instructions (Amersham, Buckinghamshire, UK).

### Statistical analysis

Treatment effect was statistically evaluated using the mean score result (i.e. percentage of stained nuclei) for each treatment group (i.e., control, sodium butyrate, tributyrin or various concentrations). To test for significant differences the one-way ANOVA test was performed. To determine the differences between all pairs, *post hoc* analysis using the Scheffé method was applied. Values are presented in a graphical format using error bars with 95% confidence intervals (CIs). *P*-values <0.05 were considered statistically significant.

## RESULTS

### Butyrate-induced growth inhibition *in vitro*

**Figure 1:**
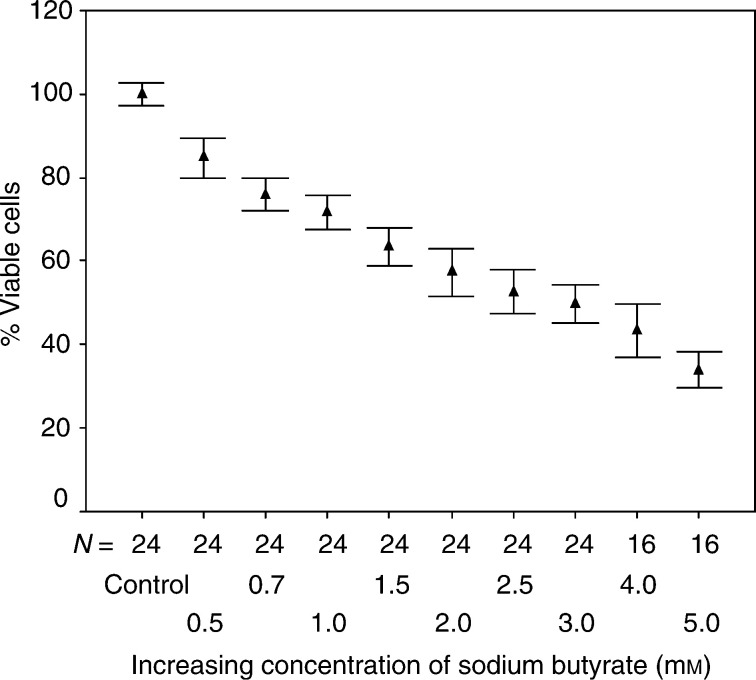
Increasing concentrations of sodium butyrate were applied on the human prostate cancer cell line PC3 starting at an initial concentration of 0.5 mM up to 5.0 mM. Growth inhibition was correlated to absorbance determined with the XTT assay. The control was set to 100%. The percentage of viable cells is given as mean values with standard deviation of repeated experiments.

Increasing concentrations of sodium butyrate and tributyrin were applied onto the cell lines PC3, TSU-Pr1 and LNCaP starting at a final concentration of 0.5 mM up to 5.0 mM. Cells were exposed for 72 h before absorbance was measured using the XTT assay. The titration curve for sodium butyrate is given in Figure 1

for the PC3 cells, demonstrating a 50% growth inhibition at about 2.5 mM final concentration. TSU-Pr1 cells were slightly less and the LNCaP cell line was slightly more sensitive to the treatment. Sodium butyrate at a dosage of 0.5 mM induced already a significant growth inhibition in all the three cell lines compared to mock-treated cells. One-way ANOVA analysis revealed a *P*-value of <0.0001. Incubation with equimolar concentrations of tributyrin revealed stronger dose-dependent growth-inhibitory effects (data not shown). This is in accordance with our previous study, where further detailed data were presented (Maier *et al*, 2000).

### Quantitative assessment of *in vivo* induction of apoptosis in prostate cancer microtumours established on the CAM

**Figure 2:**
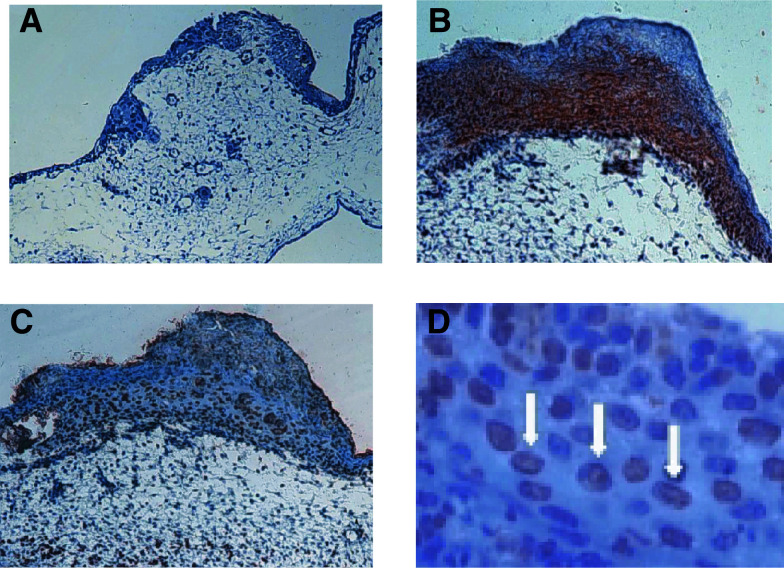
Prostate cancer microtumours growing on the CAM. (**A**) In the mock-treated control, only very few cells of the tumour, sitting on top of the CAM, show signs of apoptosis. (**B**) Strong labelling (red staining) of tumour cells by immunohistochemistry with an anti-human antibody to demonstrate the human origin of the evaluated cells (MNF 116 cytoceratin cocktail (DAKO, Germany). (**C**) Tributyrin-treated tumours growing on the CAM. TUNEL assay reveals dark brown staining as a correlate to apoptotic changes (magnification × 20). (**D**) Higher magnification of (**C**). Nuclei-bound staining by TUNEL assay is indicated by white arrows (× 800 magnification).

Tumours implanted on the CAM were examined quantitatively for apoptosis following exposure to increasing concentrations of sodium butyrate or tributyrin (0.1–5 mM) by a single i.v. injection. i.v. Injections of sodium butyrate or tributyrin revealed a strong apoptotic effect in prostate cancer microtumours over mock-treated controls as visible in the tissue sections and demonstrated in Figure 2A–D

**Figure 3:**
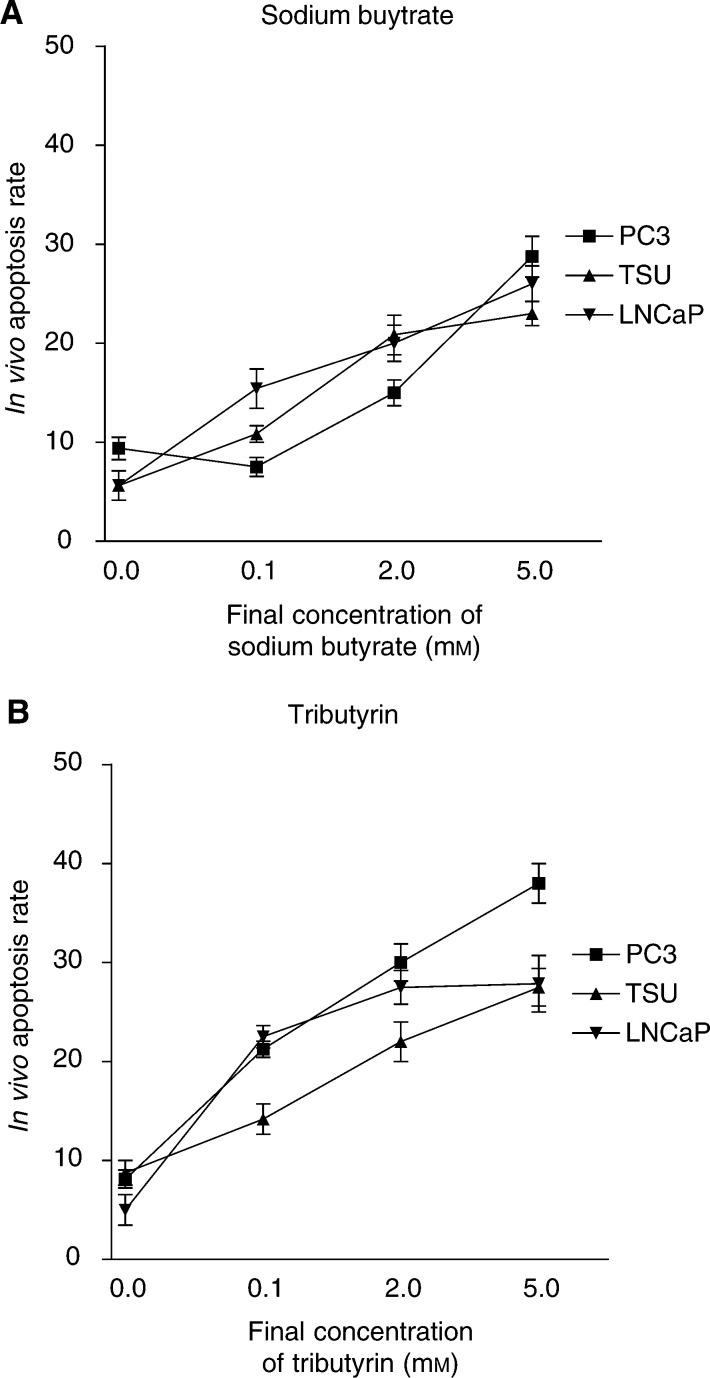
(**A**) Induction of apoptosis in LNCaP, PC3 and TSU-Pr1 human prostate cancer cells by increasing concentrations of sodium butyrate. The final *in vivo* concentration of i.v.-applied sodium butyrate varied from 0.1 to 5.0 mM. Normal saline injection served as a negative control. Apoptotic cells were assessed using the TUNEL assay. (**B**) Induction of apoptosis in the three cell lines LNCaP, PC3 and TSU-Pr1 with increasing concentrations of tributyrin.

. Both compounds, sodium butyrate and tributyrin, led to a dose-dependent increase of apoptotic cells compared to mock-treated cells. With sodium butyrate, at the maximum concentration of 5.0 mM the corresponding apoptosis rates were almost similar with 28.7±5.8, 23.0±2.7 and 26.0±5.7% of apoptotic nuclei in PC3, TSU-PR1 and LNCaP cells, respectively (Figure 3A

). When treated with tributyrin (Figure 3B) PC3 cells were the most sensitive and LNCaP cells were less sensitive. After injection of 5.0 mM tributyrin, the corresponding rate of apoptotic nuclei was 38±4.5, 27.5±5.3 and 27.8±7.5 for PC3, TSU-PR1 and LNCaP cells, respectively. Compared to a 48 h sodium butyrate exposure, the percentage of apoptotic cells induced by tributyrin exposure was higher. Interestingly, the dose–response curve of both compounds showed a substantial rate of apoptotic cells compared to mock-treated cells at already very low concentrations of 0.1 mM of sodium butyrate or tributyrin.

### Inhibition of tumour growth in the mouse model

**Figure 4:**
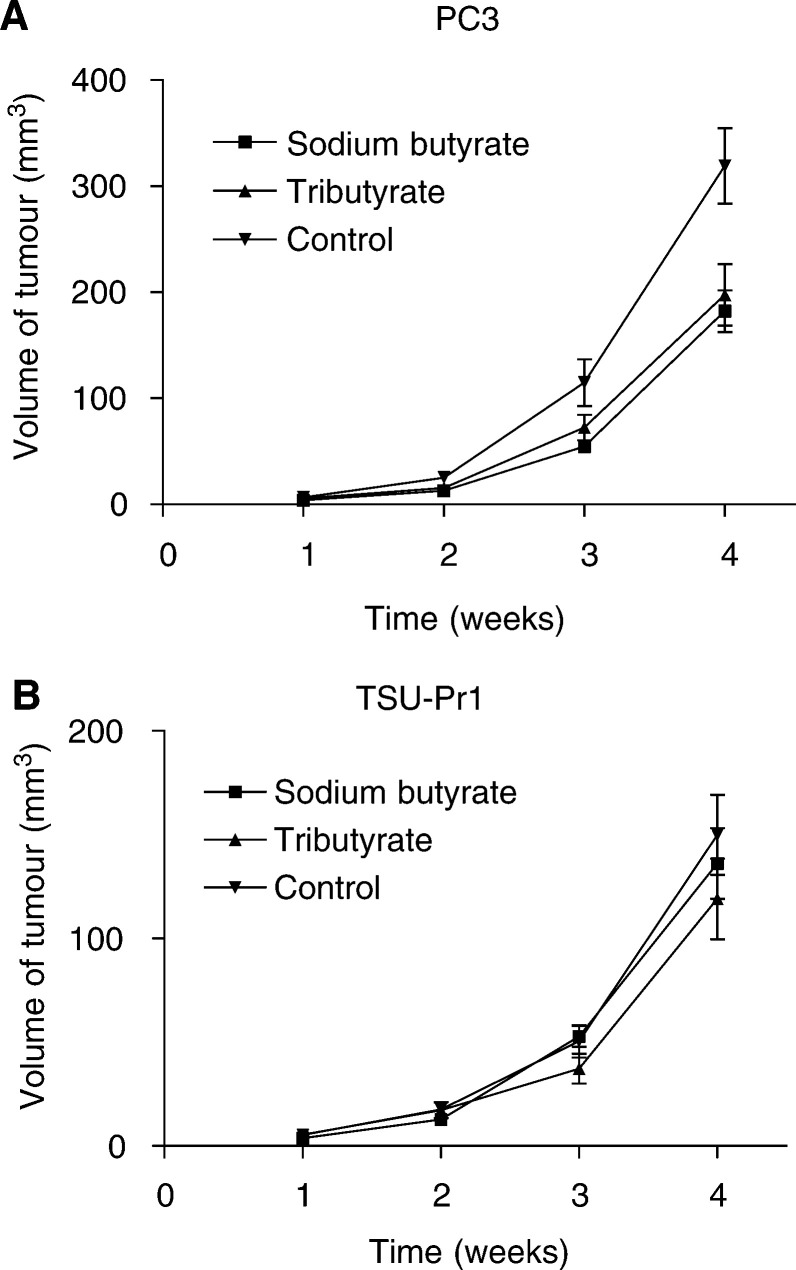
(**A**) PC3 cells were transplanted in male nude mice. Tumour volume was measured once weekly and is given as an average of 12 tumours. The mice were treated with either normal saline, sodium butyrate or tributyrin by i.p. injections with an estimated plasma concentration of 10 mM for the compounds. (**B**) Tumour volume after implantation of TSU-Pr1 prostate cancer cells in male nude mice. Same control and treatment groups as given in (**A**) for PC-3 cells.

NMR/−nu/nu male mice (7 weeks old) with an average weight of 30 g were used for implantation of human prostate cancer cells derived from the cell lines PC3 and TSU-Pr1. These cell lines were chosen for the mouse model as they easily grow without any additional condition media and form homogeneous tumours. As graphically demonstrated in Figure 4A

, the volume of the PC3 tumours was increasing over time, with the control group having the largest tumour sizes. After 4 weeks of treatment, the size of the tumours in the control group was almost double the size compared to the treatment groups. Between sodium butyrate and tributyrin treatment, there was no significant difference in tumour size throughout the observation time. With the less fast growing TSU-Pr1 cells, the tumours in the control and treatment groups were smaller. Still similar *in vivo* effects for the HDACs were observed applying the identical treatment regimen as for the PC3 cells (Figure 4B). As a parameter to reflect the general condition of the animals, the weight during the time of the experiments was documented weekly. Substantial loss of weight did not occur in any of the treatment groups during the course of the treatment.

### Ki-67 immunohistochemistry of mouse tumours

**Figure 5:**
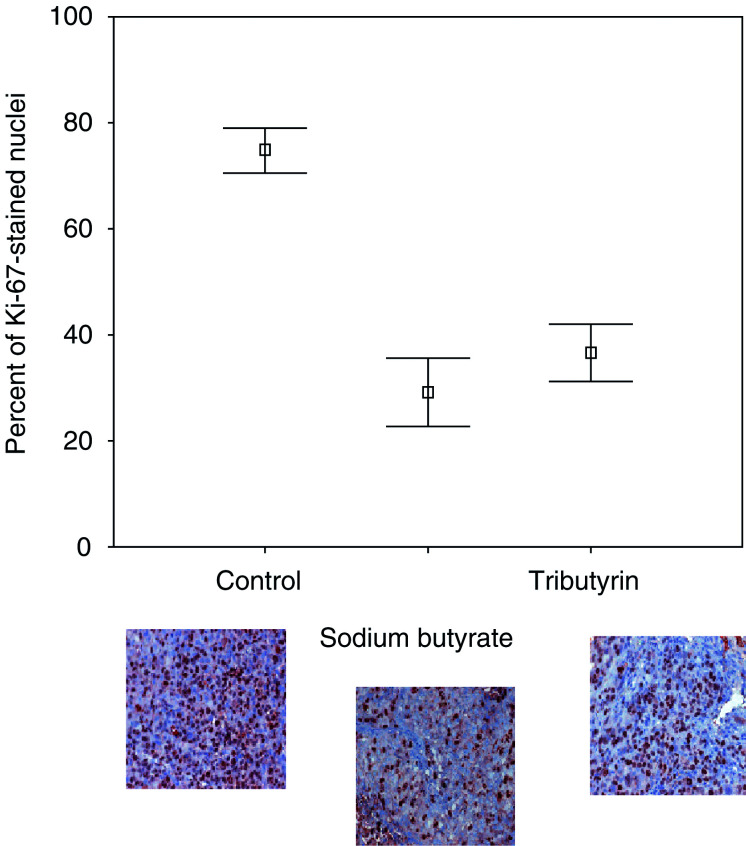
Immunohistochemistry was performed for protein expression of Ki-67. Control mouse tumours, sodium butyrate- and tributyrin-treated mouse tumours were stained with anti-human Ki-67 monoclonal antibody. Ki-67-positive cells are stained dark brown. Nuclear staining was evaluated with a computerised system. Mean percentages of positive nuclei are graphically given with 95% CI. Examples of tissue sections stained for Ki-67 for the control group, the sodium butyrate- and tributyrin-treated group are given.

Mouse tumours were removed and tissue sections were stained for Ki-67 expression in the nuclei. Control tumours, sodium butyrate- and tributyrin-treated tumours had a mean percentage of stained nuclei of 74.9 (standard error (s.e.) 2.0, 95% CI 70.6–79.1), 29.1 (s.e. 3.1, CI 22.8–35.5) and 36.7 (s.e. 2.7, CI 31.2–42.2), respectively. One-way ANOVA analysis revealed a *P*-value of <0.0001. To examine specifically the difference between the different treatment types, a *post hoc* pairwise comparison was performed. Control tumours demonstrated a significantly higher percentage of Ki-67-stained nuclei as compared to sodium butyrate-treated tumours (Scheffé method, *P*<0.0001, CI 36.5–54.9). Sodium butyrate-treated mice showed no significant different percentage of Ki-67-stained nuclei as compared to tributyrin-treated tumours (Scheffé *P*=0.14; CI –16.9 to 1.8). The percentage of Ki-67-stained nuclei for the three groups is graphically demonstrated in Figure 5

for PC3-implanted tumours. Examples of immunohistochemistry are also given, with Ki-67 staining the nuclei of dark brown cells that are in an active phase of the cell cycle.

### Expression of p21, Rb and c-myc

**Figure 6:**
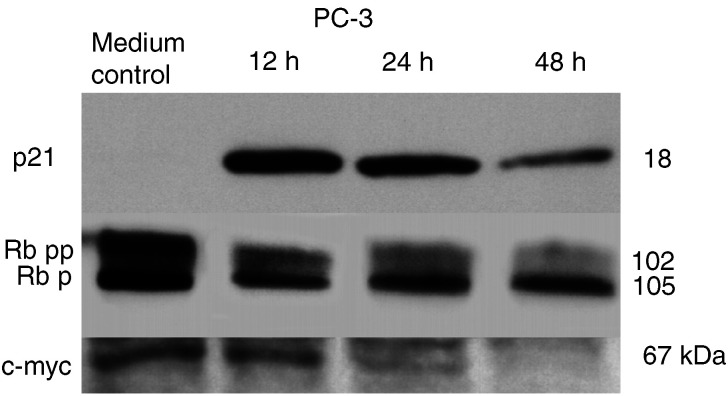
Western blot analysis using specific antibodies for detection of p21, Rb and c-myc expression. The presented blots represent expression of the targets of sodium butyrate-treated PC3 human prostate cancer cells. Expression was evaluated at several time points after treatment (internal loading control not shown). P21 expression was induced by sodium butyrate with a maximum at 12 h post-treatment. Rb expression shifted to the hypophosphorylated form in a time-dependent pattern. Simultaneously, c-myc expression decreased. Molecular weights are given in kDa.

For Western blot analysis, cells were treated with a final concentration of 5.0 mM sodium butyrate. Protein expression of the specific targets p21, Rb and c-myc, is demonstrated for PC3 cells in Figure 6

. Lysates representing different treatment times, that is, 12 and 24 and 48 h treatment, were run simultaneously on the same gel, loading equal amounts of protein. The cell cycle inhibitor p21 was not expressed in untreated PC3 (and TSU-Pr1) cells, which were both not expressing a wild-type p53. After exposure to sodium butyrate, p21 expression was induced with a maximum at 12 h post-treatment. P21 possibly could affect c-myc expression via inhibition of cyclin-CDK-dependent phosphorylation of Rb. Western blot analysis revealed an Rb-dephosphorylation after exposure to sodium butyrate in a time-dependent pattern. Simultaneously, c-myc expression decreased, supporting the hypothesis of a p21>Rb>c-myc-dependent pathway underlying the butyrate-induced growth-inhibitory effects.

## DISCUSSION

While effective surgical and radiation treatment options can be offered for clinically localised prostate cancer, advanced prostate cancer remains incurable. Androgen ablation has been the standard treatment for metastatic prostate cancer for many years. Despite an initial response to antiandrogens, disease progresses in the majority of cases independent of hormonal status. Unfortunately, the surviving cell clones are resistant to standard cytotoxic chemotherapies as well. Not surprisingly, early clinical trials have demonstrated only a very limited benefit in patients with advanced prostate cancer disease (Yagoda and Petrylak, 1993). The chemotherapy regimen was partly unsuccessful as patients had large symptomatic tumour burden, and due to their advanced age often presented with confounding comorbidities. The patients with advanced disease nowadays present a lot earlier and due to the merit of up-to-date medicine mostly with well-controlled comorbidities. These clinical aspects and increasing knowledge about biomolecular conditions of prostate cancer based on new technologies (Dhanasekaran *et al*, 2001) facilitate the identification of new chemopreventive and chemotherapeutic options for the treatment of prostate cancer. Novel agents often influence pathways that are underlying prostate cancer growth and survival. The rationale for this type of approach is the hope of increased antitumour activity with less toxicity, as those pathways are targeted that are critical for malignant but not for normal cell differentiation, proliferation and survival. One of the promising groups of drugs is the HDACs.

In our previous study, we have shown several *in vitro* effects caused by treatment of PC3, TSU-Pr1 and LNCaP cells with sodium butyrate or tributyrin (Maier *et al*, 2000). These effects included cell swelling, nuclear disruption, cell cycle arrest in the G1 phase and dose-dependent induction of apoptosis. In the XTT assay analysis in this study, the LNCaP cell line was most sensitive compared to PC3 and TSU-Pr1 cells. This could possibly be explained by the fact that LNCaP cells are known to be a cell line expressing prostatic markers and being hormone sensitive, whereas PC3 and TSU-Pr1 cells are less differentiated (Kaighn *et al*, 1979; Horoszewicz *et al*, 1983; Webber *et al*, 1996). PC3 and TSU-Pr1 cells carry molecular alterations such as a mutated p53 and the origin of TSU-Pr1 cells is discussed controversially (Iizumi *et al*, 1987; van-Bokhoven *et al*, 2001). On the chicken egg membrane, PC3 tumours seemed to be more sensitive especially when treated with tributyrin. For all cell lines it could be noted, in accordance with the reported *in vitro* observations, that sensitivity was higher when treated with tributyrin. The parameter chosen for quantification of the treatment effect was the number of apoptotic cells. This experiment demonstrates that the used butyrates show *in vivo* efficiency via the induction of apoptosis. The verification of cells of human origin was carried out by immunohistochemistry using a cytokeratin antibody cocktail. Based on the promising results of this *in vivo* model, mouse experiments were conducted for verification. In the mouse experiments we aimed for a plasma concentration of 10 mM for both drugs according to published pharmacological calculations for butyrates (Egorin *et al*, 1999). Although it could be demonstrated that relevant plasma concentrations can be achieved by oral administration (Egorin *et al*, 1999) in this study, drug application was performed by injection. Drug application by mixing the substances with the food did not prove to be effective as the pellets were partly disregarded by the animals, resulting in considerable variations of plasma levels. In comparison to the very sensitive CAM assay, in which a similar treatment effect as observed in the cell culture experiments was achieved with a single dose, a much higher total dosage was needed in the mouse model. This is most likely due to delivery and metabolism (Miller *et al*, 1987). During the course of 4 weeks, more resistant cell clones most probably do not emerge. The tumour size was significantly less increasing in the treated animals with no significant difference between sodium butyrate or tributyrin applied with equal final plasma concentration. None of the mice died due to acute toxic treatment effects and none of the animals showed major changes in the weight as a parameter for good drug tolerance. The tumour sizes correlate well with the percentage of Ki-67-stained nuclei as observed by immunohistochemistry and computerised objective evaluation. In this study, the Ki-67-positive cells were significantly less in the treated group compared to the control group of untreated mice. Increased differentiation induced by butyrates has been shown earlier in *in vitro* experiments using the LNCaP cell lines (Carducci *et al*, 1996).

The given immunoblot results suggest that the mechanism for a treatment effect by butyrates is independent of a wild-type p-53 status (Carducci *et al*, 1996). Overexpression of p21 has been shown to activate Rb (Agarwal *et al*, 1998; Keegan *et al*, 1998). Rb is known to be functionally active in an underphosphorylated conformation and is inactivated during the late G1 phase by cyclin-dependent phosphorylation, allowing the cells to proceed from G1 to S phase (Zhao *et al*, 1997). The given results suggest that an intact p21, Rb and c-myc may have to be present for treatment effect. A similar observation has been made for a novel HDAC inhibitor FK228 (Sasakawa *et al*, 2003). Still, the given data obviously look only at a very small portion of possibly involved pathways and thus have to be considered as preliminary. It cannot be ruled out that the observed changes could be a parallel issue as for example growth inhibition, induced by sodium butyrate, has been described in a cell line that has abrogated Rb function (Rashid *et al*, 2001). There might be other and potentially even more important structures such as cyclin A and D, p27 and protein kinase C, which are involved in the process of butyrate-induced growth inhibition (Chen *et al*, 2003; Kim *et al*, 2003; Siddiqui *et al*, 2003). Nevertheless, exploring the activation of p-21, including the role of Sp1 transcription factor, is a very promising approach (Wang *et al*, 2000; Rashid *et al*, 2001; Margueron *et al*, 2003), as this gene appears to be rarely mutated in common human malignancies in contrast to p53 (Terao *et al*, 2001). A similar pattern of mechanism has been described for several cell lines including prostate cancer cell lines for the effect of phorbol ester (Blagosklonny *et al*, 1997). This observation possibly suggests that a combination of phorbol ester and butyrates may have an additive effect on tumour cells (McBain *et al*, 1996; Rahmani *et al*, 2002).

In contrast to the majority of solid tumours, prostate cancer is a very heterogeneous tumour, which is obvious for example in the various Gleason patterns represented in one gland (Gleason, 1966). New technologies such as laser microdissection help to gain an insight into the underlying patho-morphologies of distinct tumour cells (Rubin, 2002). This may aid in understanding the complexity of intervening pathways of prostate cancer growth. From this understanding, it is likely that monotherapy might not be sufficient for treatment but rather a combination of drugs targeting various mechanisms. Based on the presented *in vivo* results, butyrates are very promising agents in prostate cancer control independent of hormone sensitivity. The anticancer effects might be supported by a combination with other drugs and a combined treatment approach may offer the chance to a reduced dosage with less side effects (Newmark and Young, 1995; Conley *et al*, 1998; Halicka *et al*, 2000; Banwell *et al*, 2003).

In this study, we have demonstrated a substantial *in vivo* treatment effect, which can be induced by the application of sodium butyrate or the orally applicable tributyrin in human prostate cancer. These two HDACs have shown a strong cell growth-inhibitory and proapoptotic activity. These effects seem to be independent of p53 status and a pathway including p21, Rb and c-myc is possibly involved in these effects. The given *in vivo* results may provide the rationale to apply these HDACs in well-controlled clinical trials as ‘gene-regulating chemoprevention’ in patients being at high risk of recurrence after specific therapy or as ‘gene-regulating chemotherapy’ in a combination regimen for patients with locally or distant advanced prostate cancer.
